# Cardiovascular risk in patients with bipolar disorder in Tunisia

**DOI:** 10.1192/j.eurpsy.2023.1456

**Published:** 2023-07-19

**Authors:** D. Njah, M. Lagha, S. Boudriga, W. Homri, I. Ben romdhane, R. Labbene

**Affiliations:** psychiatry “C”, Razi hospital, tunis, Tunisia

## Abstract

**Introduction:**

Bipolar disorder (BD) is a multisystemic disorder affecting not only thymic regulation but also immunologic function and cardiovascular status. In fact , BD itself appears to confer risk for cardiovascular disease independent of treatments used to manage the disorder, which results in an increase in risk of morbidity and mortality compared to the general population. Indeed, according to the literature, the life expectancy of patients with BD is reduced by eight to ten years, and particularly, cardiovascular events are two to three times more frequent and occur earlier.

**Objectives:**

The objectives of our study were to determine the prevalence of cardiovascular risk factors in patients with BD in remission and to compare it to a control sample.

**Methods:**

This was a case-control study that took place over an 18-month period, from January 2, 2020 to June 30, 2021, in RAZI hospital, in Tunisia . Statistical analysis was performed by SPSS 26.0.

**Results:**

Sixty patients in remission and sixty healthy controls were included in this study.

The mean age was 42.5 ± 11.1 years with extremes of 20 and 60 years in the case group, while the mean age was 42.7 ± 10.2 years with extremes of 20 and 63 years in the control group.

At least one cardiovascular risk factor was found in 91% of patients with BD vs 78% of controls, and 92% of patients were smokers vs 68% of controls, with a significant difference between the two groups (p=0.041 and p=0.001), respectively.

**Conclusions:**

Given the high risk of cardiovascular disease, rigorous cardiovascular risk assessment is critical for patients with BD. Psychiatrists should be aware of this problem and carefully monitor these patients for cardiovascular risk factors, including smoking, as part of their standards of care.

**Disclosure of Interest:**

None Declared

